# Nanohybrid structure analysis and biomolecule release behavior of polysaccharide-CDHA drug carriers

**DOI:** 10.1186/1556-276X-8-417

**Published:** 2013-10-08

**Authors:** Li-Ying Huang, Ting-Yu Liu, Tse-Ying Liu, Andreas Mevold, Andri Hardiansyah, Hung-Chou Liao, Chin-Ching Lin, Ming-Chien Yang

**Affiliations:** 1 Department of Materials Science and Engineering, National Taiwan University of Science and Technology, Taipei 106, Taiwan; 2 Department of Materials Engineering, Ming Chi University of Technology, New Taipei City 24301, Taiwan; 3 Institute of Biomedical Engineering, National Yang-Ming University, 155, Sec. 2, Lih-Nong St, Taipei 112, Taiwan; 4 Material and Chemical Research Laboratories, Industrial Technology Research Institute (ITRI), Hsinchu 31040, Taiwan

**Keywords:** Chitosan, Ca-deficient hydroxyapatite (CDHA), Drug release, Nanohybrids

## Abstract

Nanoscaled polymer composites were prepared from polysaccharide chitosan (CS) and Ca-deficient hydroxyapatite (CDHA). CS-CDHA nanocomposites were synthesized by *in situ* precipitation at pH 9, and the CS-CDHA carriers were then fabricated by ionic cross-linking methods using tripolyphosphate and chemical cross-linking methods by glutaraldehyde and genipin. Certain biomolecules such as vitamin B_12_, cytochrome c, and bovine serum albumin were loaded into the CS-CDHA carriers, and their release behaviors were investigated. Furthermore, these CS-CDHA carriers were examined by transmission electron microscopy, electron spectroscopy for chemical analysis, and X-ray diffraction. The release behavior of the biomolecules was controlled by the CS/CDHA ratios and cross-linked agents. By increasing the concentration of CS and the concentration of the cross-linking agents, cross-linking within carriers increases, and the release rate of the biomolecules is decreased. Moreover, the release rate of the biomolecules from the CS-CDHA carriers at pH 4 was higher than that at pH 10, displaying a pH-sensitive behavior. Therefore, these CS-CDHA hydrogel beads may be useful for intelligent drug release and accelerate bone reconstruction.

## Background

Recently, much attention has been focused on chitosan (CS)-based hydrogel for cartilage tissue engineering and bone substitute with controlled release function due to its structure similar to that of natural glycosaminoglycan [1-3]. CS is a cationic polysaccharide with an isoelectric point of 6.2 [4], thus is pH sensitive and has been proposed for electrically modulated drug delivery [5]. Furthermore, CS has been identified as hydrophilic, non-toxic, biodegradable, antibacterial, and biocompatible. In our previous study [6], we demonstrated that the addition of clay to the CS matrix could strongly affect the cross-linking density as well as the mechanical properties, swelling-deswelling behavior, and fatigue property of the nanohybrids. Hence, the incorporation of negatively charged delaminated (exfoliated) montmorillonite is expected to electrostatically interact with the positively charged -NH_3_^+^ group of CS to generate a strong cross-linking structure in the nanohydrogel [7], thus strongly affect the macroscopic property of the nanohydrogel and the drug diffusion through the bulk entity.

There have been some reports in the preparation of CS nanoparticles by ionic and chemical cross-linking methods, for example, the use of an ionic gelation method to prepare CS NPs as reported by Calvo et al. [8]. Cationic CS nanoparticles were formed through the inter- and intra-cross-linking of the amino groups of CS with the negatively charged phosphate groups of tripolyposphate (TPP). TPP is a non-toxic polyanion which can interact with CS via electrostatic forces to induce ionic cross-linked networks [9], which could be used for the preparation of CS hydrogel beads owing to its immediate gelling ability. Furthermore, Mi et al. [10] reported the preparation of chitosan gel using a natural chemical cross-linker, i.e., genipin (GP), which is obtained from its parent compound traditionally used as a component of Chinese medicine, geniposide, which was separated from *Gardenia jasminoides* Ellis. GP cross-linked networks in the biopolymers display significantly less cytotoxicity than those chemical cross-linked by glutaraldehyde (GA) and thus recently was widely used for various biomedical applications [11-14].

The purpose in this study is to modulate the release rate of biomolecules from highly swollen hydrogel beads and its loose structure [15] in order to extend the drug release period of the CS hydrogel. The drug release permeability of CS can be further regulated by the incorporation of Ca-deficient hydroxyapatite (Ca_10-*x*_(PO_4_)_6-*x*_ (HPO_4_)_*x*_(OH)_2-*x*_, 0 ≤ *x* ≤ 1, CDHA, Ca/P = 1.5) nanorods, because it has long been employed to improve the mechanical strength and osteoconductivity of chitosan [16-18]. The influence of the nanofiller (CDHA nanorods) in the CS hydrogel for the drug release behavior might be critical and can be explored further.

Therefore, the major research objective of this study is to explore the role of CDHA nanorods in the release behavior of biomolecules (vitamin B_12_, cytochrome c, and bovine serum albumin (BSA)) from CS hydrogel beads. In addition, the degree and methods (ionic or chemical) of cross-linking in the CS hydrogel beads were also investigated. This study is expected to provide a fundamental understanding of the CS-CDHA nanocomposite drug carrier used for medical applications and also of the drug (growth factor) delivery to enhance bone repair.

## Methods

### Synthesis of CS-CDHA nanocomposites

CS-CDHA nanocomposites with various CDHA contents were prepared via *in situ* processes to characterize the influence of nanofiller and polymer-filler interaction on the behavior of this drug delivery system. Chitosan (molecular weight 215 kDa, 80% degree of deacetylation) was purchased from Sigma-Aldrich (St. Louis, MI, USA). CS solution (1% (*w*/*v*)) was first prepared by dissolving the CS powder in 10% (*v*/*v*) acetic acid solution. For the *in situ* process (PO_4_^3-^→CS→Ca^2+^), H_3_PO_4_ aqueous solution (0.167 M) was first added into the CS solution, and Ca(CH_3_COO)_2_ aqueous solution (0.25 M) was then added into this mixture solution under stirring for 12 h. The pH value was kept at 9 by adding NaOH solution (1 M). The nanocomposites with different volume ratios of CS/CDHA were modulated at 0/100, 10/90, 30/70, 50/50, 70/30, and 100/0, abbreviated as CDHA, CS19, CS37, CS55, CS73, and CS, respectively. Subsequently, these CS-CDHA nanocomposites were dried at 65°C for 24 h.

### Preparation of CS-CDHA hydrogel beads

Various ratios of CS/CDHA nanocomposites and biomolecules (vitamin B_12_, 1,355 Da; cytochrome c, 12,327 Da; or BSA, 65,000 Da) were dissolved in the 10% (*v*/*v*) acetic acid solution and then the mixing solution was dropped into the different concentrations of TPP (1, 5, 10 wt.%) for ionic cross-linking or further chemical cross-linking by GA or GP under stirring. The morphology of the CS-CDHA carriers (diameter 500 to 1,000 μm) was evaluated using an optical microscope (OM).

### Characterization

The crystallographic phase of the CDHA/CS nanocomposites was identified by X-ray diffraction (XRD, M18XHF, Mac Science, Tokyo, Japan). The morphology of the CDHA nanocrystals and various CS-CDHA nanocomposites were observed by transmission electron microscopy (TEM, JEOL-2000FX, Tokyo, Japan). The chemical structure change was evaluated by electron spectroscopy for chemical analysis (ESCA), equipped with MgKα at 1,253.6 eV and 150 W power at the anode. A survey scan of the varying electron volts for N_1*s*_, Ca_2*p*_, and P_2*p*_ was taken.

### Drug release test

These nanocomposite hydrogel beads were put into phosphate-buffered solution (pH 7.4) to test for drug release. The release medium was withdrawn for each juncture and replaced with equivalent volume of fresh buffer. UV-visible spectroscopy (Agilent 8453, Agilent Technologies Inc., Santa Clara, CA, USA) was used for the characterization of absorption peak to determine the amount of vitamin B_12_ (361 nm), cytochrome c (410 nm), or BSA (562 nm, using BCA kits) released via the use of predetermined standard concentration-intensity calibration curve.

The drug release percent was determined using Equation (1) [19]:

(1) $$\mathrm{Cumulative}\;\mathrm{release}\left(\%\right)=R_{t}/L\times 100\%,$$

where *L* and *R*_t_ represent the initial amount of drug loaded and the cumulative amount of drug released at time *t*, respectively.

## Results and discussion

**Figure 1:**
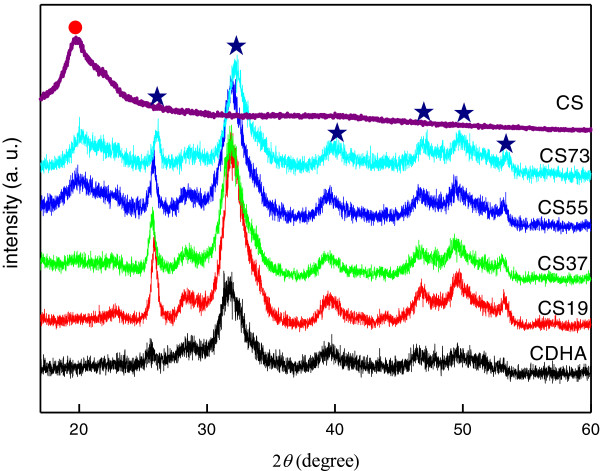
**XRD patterns of pristine CS, pristine CDHA, and various CS-CDHA nanocomposites.** Red circle: peak of CS; blue star: peak of CDHA.

**Figure 2:**
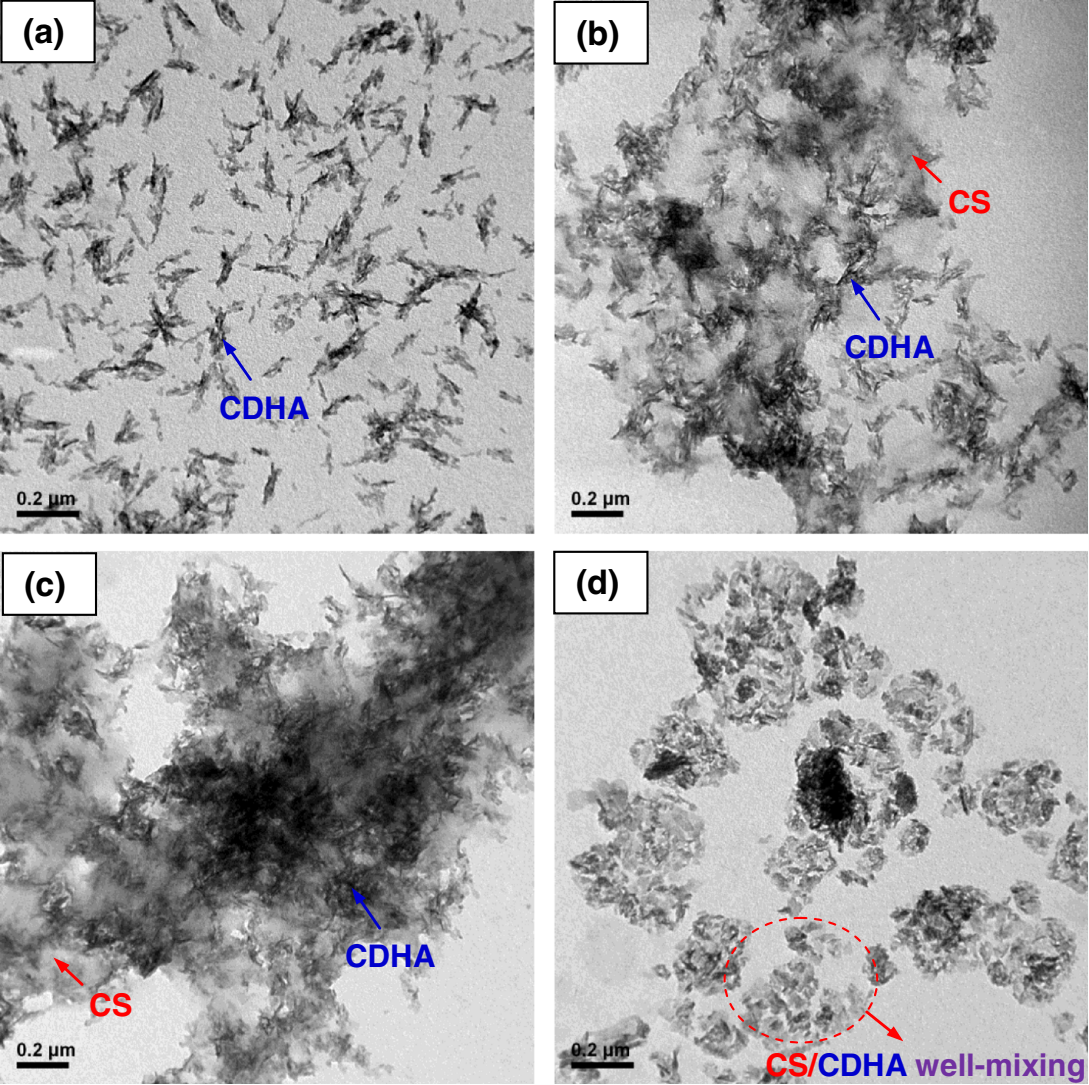
**TEM images of CS-CDHA nanocomposites. (a)** Pristine CDHA, **(b)** CS37, **(c)** CS55, and **(d)** CS73 nanocomposites.

**Figure 3:**
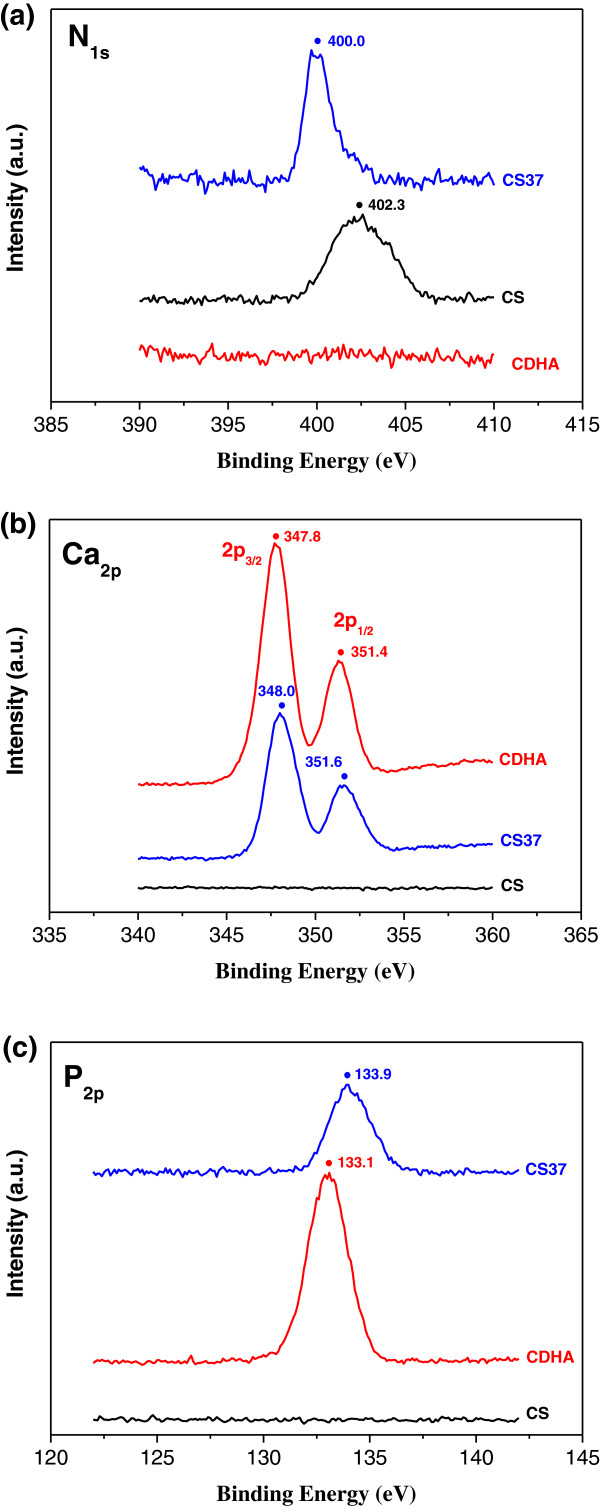
**ESCA spectra of CS-CDHA nanocomposites. (a)** N_1*s*_, **(b)** Ca_2*p*_, and **(c)** P_2*p*_ for pristine CS, pristine CDHA, and CS37 nanocomposites.

The CS-CDHA nanohybrids were prepared using ionic gelation. At first, H_3_PO_4_ solution was adsorbed on the CS matrix and then Ca(CH_3_COO)_2_ solution (PO_4_^3-^→CS→Ca^2+^) was added. In this *in situ* precipitated method, CDHA nanorods were encapsulated within polysaccharide CS matrix, resulting in a nanocomposite with homogeneous nanostructure. At pH 9, the nanohybrids (CS and CDHA nanocrystals) were observed. The CDHA nanorods were incorporated into the CS polymer network homogeneously, as shown in the XRD (Figure 1) pattern, TEM (Figure 2), and ESCA (Figure 3).

Figure 1 shows the XRD patterns of the CDHA, CS, and CS-CDHA nanocomposites. One major peak at 26° and 32°, and four minor peaks at 40°, 46°, 50°, and 53° were observed (peak of pure CS appeared at 21°). According to the ICDD No. 39–1894 and No. 46–0905, these peaks could be identified as semi-crystalline of CS (2*θ* approximately 21°) and crystalline of CDHA, respectively. Using CS73 nanocomposite as an example, both CS and CDHA characteristic peaks (seven peaks) were observed. This indicated that the CDHA/CS nanocomposites could be synthesized via *in situ* precipitated processes. Furthermore, it was observed that the crystallinity of CS decreased with increasing CDHA content, because the homogenously dispersive CDHA nanorods would induce defects in the CS network. Moreover, it is also demonstrated that strong polymer-filler interaction could modify the molecular configuration of the polymer chains in the vicinity of the filler to the formation of localized amorphous regions. This would inhibit and retard the crystalline development of the CS chains. It became more pronounced when the CDHA content exceeds 30 wt.%. However, the crystallinity of CDHA seems to be enhanced by the addition of CS. The full-width at half maximum of the XRD peak of the CS-CDHA nanocomposites was observed to be lower than that of the pristine CDHA, thereby displaying sharper peak (better crystallinity). Thus, we suggest that the CS chains might induce the crystallinity of CDHA.

Figure 2 shows the TEM images of the pristine CDHA (a), CS37 (b), CS55 (c), and CS73 (d) nanocomposites. The pristine CDHA exhibited needle-like structure of nanorods (5 to 20 nm in diameter and 50 to 100 nm in length). The CS-CDHA nanocomposites exhibited homogenously dispersed nanorods in the CS networks, especially in the CS73, as illustrated in Figure 2b,c,d. The reason is that the electrostatic attraction between the NH_3_^+^ group (positive charge of the CS chains) and the PO_4_^3-^ group (negative charge of the CDHA nanorods) served as the stable force for the colloid suspension, favoring the dispersion of CDHA. Moreover, the structure of the CS-CDHA nanocomposites (CS73) became denser with the increase of the CS content due to the better compatibility and stable network of high molecular weight of CS. In contrast, CS55 and CS37 exhibited less dense morphologies.

A comparison of the chemical binding energy change of the pristine CDHA, pristine CS, and CS37 nanocomposites was shown in the ESCA spectra. The ESCA analysis shows that the surface was mainly composed of N, Ca, and P atoms, which could represent the chemical structure and interaction of CS (N atom) and CDHA (Ca and P atoms). Figure 3a shows the ESCA data of N_1*s*_ scan spectra in CS, CDHA, and CS37. The N_1*s*_ peak in the pristine CS was found at 402.3 eV, implying the amino group of CS (no peak existing in the pristine CDHA). However, the NH_2_ peak was shifted from 402.3 to 400.0 eV in the CS37, implying the complex formation of CS and CDHA. Two Ca_2*p*_ peaks of the pristine CDHA were observed with the binding energy of 347.8 eV (2*p*_3/2_) and 351.4 (2*p*_1/2_), as indicated in Figure 3b. Two peaks (2*p*_3/2_ 348.0 eV and *2*p_3/2_ 351.6 eV) were exhibited in CS37 and displayed 0.2 eV chemical shift compared to the pristine CDHA, suggesting the formation of CDHA in the CS37 and some chemical interaction between CS and CDHA (no additional peak in the pristine CS). Similar with the ESCA spectrum of Ca_2*p*_, 0.8 eV (133.1-eV shift to 133.9 eV) chemical shifts were found between the pristine CDHA and CS37 in the P_2*p*_ spectrum. These results indicate that the CDHA nanorods were grown in the CS matrix through *in situ* precipitated process.

**Figure 4:**
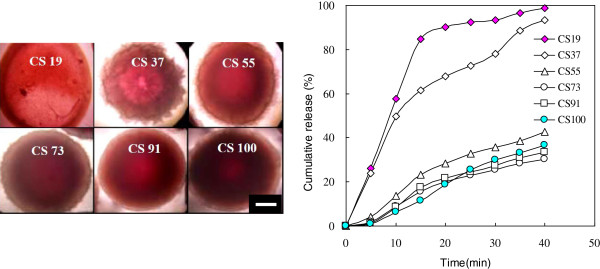
**OM photos and vitamin B**_**12 **_**cumulative release (%) of various CS/CDHA nanocomposites hydrogel beads.** TPP 10%, scale bar = 200 μm.

When it was fully ionic cross-linked by P_3_O_10_^3-^ ions in the TPP solution, CS with positive charge (NH_3_^+^) would consume most of the binding sites and interact with the negatively charged phosphate groups of TPP. The model biomolecules were encapsulated into the CS-CDHA carriers (hydrogel beads) to evaluate their suitability as a delivery system. Figure 4 show the OM images of the CS-CDHA carriers of the pristine CS and various ratios of CS-CDHA nanocomposites cross-linked by 10% TPP (diameter 500 to 1,000 μm). With the increase of CS, the hydrogel beads exhibited more stable and denser chemical structure, showing higher cross-linked density by TPP and thicker wall of beads (dark and black corona). It exhibits very loose structure in CS19, but dense morphology in CS91. The cumulative release rate (vitamin B_12_) of these CS-CDHA nanocomposites is in the order of CS19 > CS37 > CS55 > pristine CS > CS91 > CS73. CS73 showed the lowest drug cumulative release because it has the highest compact structure, as shown in the TEM image (Figure 2). We suggest that CDHA might play an important role, limiting the path of drug release in a suitable addition ratio of CDHA.

**Figure 5:**
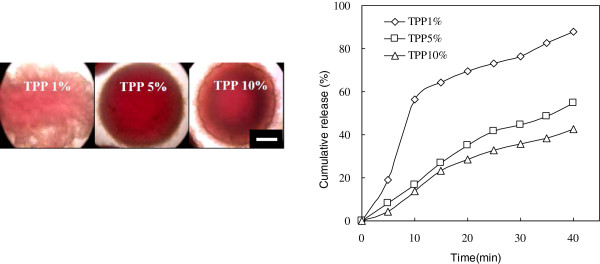
**OM photos and vitamin B**_**12 **_**cumulative release (%) of CS55 hydrogel beads.** The beads are ionically cross-linked by TPP 1%, TPP 5%, and TPP 10%. Scale bar = 200 μm.

**Figure 6:**
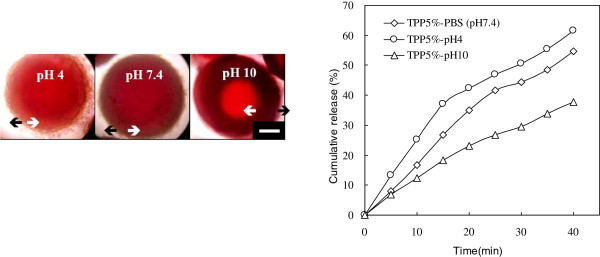
**OM photos and vitamin B**_**12 **_**cumulative release (%) in pH 4, pH 7.4, and pH 10.** CS55 hydrogel beads, TPP 5%; scale bar = 200 μm.

Figure 5 shows the effect of the ionic cross-linker (TPP) concentration for drug (biomolecules) release. The result indicates that higher concentration of TPP would cause the lowering of drug release due to the stronger network of the hydrogel beads. Stable hydrogel beads were difficult to form with 1% TPP due to weak cross-linkage. Furthermore, pH-sensitive behavior was found in the CS-CDHA nanocomposite by its polyelectrolyte complex nature. The CS polymer chains would swell and expand at pH below 6.2 (isoelectric point of chitosan is 6.2) but deswell and shrink at pH above 6.2. Thus, rapid release of CS55 hydrogel beads was observed at pH 4, while slow release occurred at pH 10 (Figure 6). The OM image of hydrogel beads at pH 10 displayed thicker corona wall; thus, drug release is slowest at pH 10.

**Figure 7:**
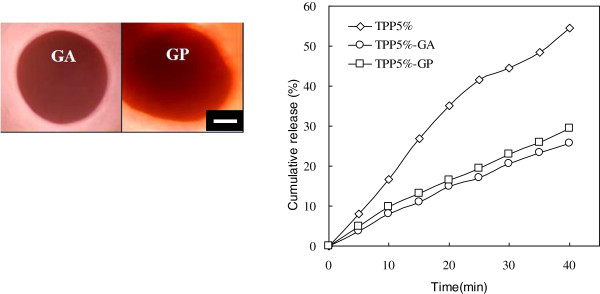
**OM photos and vitamin B**_**12 **_**cumulative release (%) of chemical cross-linking CS55 hydrogel beads.** The beads are chemical-cross-linked by GA and GP after TPP 5% ionically cross-linked by TPP. Scale bar = 200 μm.

In order to achieve sustained release behaviors, the chemical cross-linkers (GA and GP) were used to increase the density and strength of cross-linking in the CS-CDHA carriers. Figure 7 demonstrated that GA-cross-linked hydrogel beads display the slowest release rate. The result suggests the capability of cross-linking using GA is better than those using GP and TPP. However, GA is toxic to human bodies, which would generate some side effects. In contrast, GP is a nature cross-linker (non-cytotoxic), which is a good candidate for modified CS-CDHA carriers. The reacting time of GP is very slow; therefore, the CS-CDHA carriers need to be cross-linked by TPP first to solidify the structure before reacting with GP. This is the optimum process to achieve the sustained release purpose.

**Figure 8:**
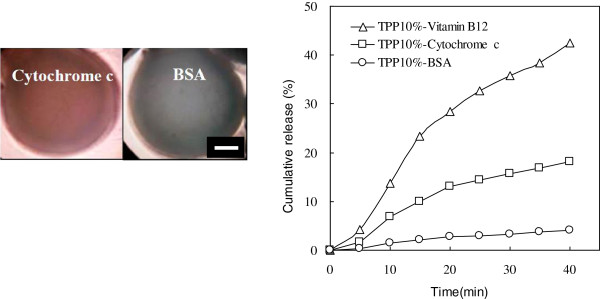
**OM photos and cumulative release (%) of vitamin B**_**12**_**, cytochrome c, and BSA in CS55 hydrogel beads.** TPP 10%, scale bar = 200 μm.

Finally, the comparison of the different molecular weight effects of biomolecules was investigated. Figure 8 shows that the slower drug release occurred in larger biomolecules, displaying in the order of BSA (65, 000 Da) < cytochrome c (12,327 Da) < vitamin B_12_ (1,355 Da). The result illustrated that the rate of drug release would be changed with different sizes of biomolecules due to the pore-size barrier of the CS-CDHA carriers. Therefore, a suitable drug carrier would be anticipated to fabricate for various sizes of biomolecules (such as growth factors and therapeutic drugs) to achieve the sustained release for biomedical applications.

## Conclusion

**Figure 9:**
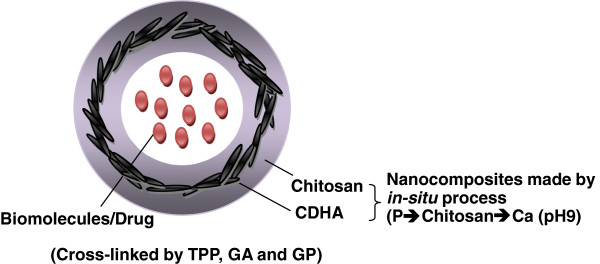
**Novel chitosan/Ca-deficient hydroxyapatite nanocomposite via an *in situ *** **precipitation process at pH 9.**

Novel biocompatible hybrid nanocomposites consisting of chitosan and CDHA were successfully synthesized via an *in situ* precipitation process at pH 9 (Figure 9) for drug delivery purpose. CS/CDHA nanocomposites were then cross-linked into hydrogel beads by tripolyphosphate, glutaraldehyde, and genipin, respectively. Various biomolecules could be encapsulated in the beads and exhibit different release behaviors. Experimental results show that the drug release kinetics of the CS-CDHA carriers was affected by the incorporation of CDHA nanoparticles. The slowest release rate was observed in CS73 (30% CDHA addition) due to its more stable structure and smaller pore size. Therefore, CDHA nanocrystal can simultaneously function as a bioactive filler and drug release regulator. The drug release rate of biomolecules also could be modulated by cross-linked agent. The application of GA will produce the densest structures, leading to the slowest drug release of biomolecules. These CS-CDHA carriers also exhibited pH-sensitive behavior. It displayed faster release rate at pH value of 4 and slowest release rate at pH value of 10, due to swelling behavior of CS at pH 4. It might provide valuable information for a better design of chitosan hybrids for drug-loaded implant with improved bioactivity and controlled drug release function. Furthermore, chitosan-CDHA nanocomposite drug carriers with pH-sensitive property which can lead to intelligent controlled release of drugs can be used as gastric fluid-resistant drug vehicles and for bone repair.

## Competing interests

The authors declare that they have no competing interests.

## Authors' contributions

LYH, TYuL, TYiL, and MCY had conceived and designed the experiments. LYH, AH, and TYuL performed the experiments. AM, AH, TYiL, HCL, and CCL contributed ideas and material analyses. LYH, TYuL, AM, and MCY wrote the manuscript. All authors read and approved the final manuscript.

## Authors' information

LYH is a postdoctoral fellow at the National Taiwan University of Science and Technology. TYuL holds an assistant professor position at Ming Chi University of Technology. AH and AM are PhD students at the National Taiwan University of Science and Technology. TYiL holds an assistant professor position at the National Yang-Ming University. HCL and CCL are researcher and manager at Industrial Technology Research Institute (ITRI) of Taiwan, respectively. MCY holds a professor position at the National Taiwan University of Science and Technology.
